# Hepatocyte Growth Factor Levels in the Saliva and Gingival Crevicular Fluid in Smokers with Periodontitis

**DOI:** 10.1155/2014/146974

**Published:** 2014-10-15

**Authors:** Sukumaran Anil, Sajith Vellappally, R. S. Preethanath, Sameer A. Mokeem, Hani S. AlMoharib, Shankargouda Patil, Elna P. Chalisserry, Abdulaziz A. Al Kheraif

**Affiliations:** ^1^Department of Periodontics and Community Dentistry, College of Dentistry, King Saud University, P.O. Box 60169, Riyadh 11545, Saudi Arabia; ^2^Dental Biomaterials Research Chair, Dental Health Department, College of Applied Medical Sciences, King Saud University, Riyadh 11433, Saudi Arabia; ^3^Department of Oral Pathology and Microbiology, Faculty of Dental Sciences, M. S. Ramaiah University of Applied Sciences, Bangalore, Karnataka, India; ^4^College of Dentistry, King Saud University, P.O. Box 60169, Riyadh 11545, Saudi Arabia

## Abstract

Hepatocyte growth factor (HGF) production by oral fibroblasts is enhanced by various molecules that are induced during inflammatory conditions including periodontitis. HGF plays an important role in the progression of periodontitis, by stimulating intense growth of epithelial cells and preventing regeneration of connective tissue attachments. Smokers have a greater risk factor in the pathogenesis and progression of periodontal disease. The objective of the study was to estimate the level of HGF in saliva and gingival crevicular fluid (GCF) in smokers with periodontitis and to compare these levels with that of nonsmokers with periodontitis and healthy controls. The HGF levels were found to be significantly high in the saliva and GCF of smokers with periodontitis compared to both never-smokers with periodontitis and the healthy control group. The elevated levels of HGF in the saliva and GCF in the study population could explain the intrinsic mechanism triggering the severity of the periodontitis in smokers. Further studies are necessary to validate the current observations and to establish a sensitive marker to predict periodontal disease activity.

## 1. Introduction

Cigarette smoking is a significant risk factor in the pathogenesis and progression of periodontal disease [1, 2]. Smokers have a greater risk of more extensive and severe alveolar bone loss. Studies have shown that smoking impairs various aspects of the innate and adaptive immune responses, including altered neutrophil function, antibody production, altered fibroblast activity, vascular factors, and inflammatory mediator production [3–10]. Following initiation of periodontal disease, host inflammatory cells are recruited, and inflammatory cytokines, such as IL-1*β*, IL-6, and TNF-*α*, are released from fibroblasts, macrophages, connective tissue, and junctional epithelial cells. Consequently, host-derived enzymes, such as MMP-8, MMP-9, and calprotectin, are released by polymorphonuclear leukocytes (PMNs) and osteoclasts, leading to the degradation of connective tissue and alveolar bone [11].

Hepatocyte growth factor (HGF), a protein secreted by mesenchymal cells, regulates angiogenesis, vascular permeability, cell migration, reepithelialization, and other wound healing processes [12]. HGF is known to be a multifunctional cytokine involved in a variety of physiological processes, including tissue development, regeneration, and wound healing [13]. HGF plays an important role in the progression of periodontitis, by stimulating intense growth of epithelial cells and preventing regeneration of connective tissue attachments. HGF is a well-known serum marker for various diseases, including periodontitis [14]. An association between HGF and periodontitis has been previously reported [15, 16]. The HGF concentration in the gingival crevicular fluid (GCF) of patients with periodontitis is approximately 10-fold higher than that of healthy subjects [17]. High levels of HGF in GCF have been observed at periodontally compromised sites [18, 19].

Traditional clinical measurements, such as probing pocket depth, bleeding on probing, and clinical attachment loss, are used for the diagnosis of periodontal disease and are not often useful because they indicate previous periodontal disease rather than current disease activity [20]. Knowing the disease activity might help in early intervention in patients with the disease. Although several epidemiological cross-sectional studies have found an association between periodontitis and smoking, knowledge of the mechanisms involved in the severity of periodontitis in smokers is scarce. Hence, in the present study, the saliva and gingival crevicular fluid HGF levels were estimated to assess periodontal disease activity in smokers and to compare these levels with those present in a nonsmoking group of patients with periodontitis.

## 2. Materials and Methods

### 2.1. Study Population

A total of 90 systemically healthy, smoking, and nonsmoking individuals (30 participants in each group), between 25 and 50 years old, were enrolled in this study from November 2011 to March 2012. Individuals included in the test groups were selected from patients who were referred to the periodontology clinics for the diagnosis and treatment of periodontitis. The control group (*n* = 30) was derived from those individuals who attended restorative dental clinics and from the staff and interns at the College of Dentistry. The periodontal status of the patients and control group was assessed according to the classification of the American Academy of Periodontology [21]. Smoking status was determined based on daily consumption [22]. Approval from the ethics committee was obtained from the College of Dentistry Research Center (CDRC), King Saud University, Riyadh, Saudi Arabia.

Sixty patients, with a periodontal probing depth ≥4 mm and clinical attachment loss ≥2 mm in at least 30% of their teeth, were diagnosed as the chronic periodontitis group. Individuals who smoked a minimum of 20 cigarettes per day for more than two years were included in the smoker periodontitis group (*n* = 30). Individuals who used other forms of smoking, along with cigarettes, were excluded from this study. The remainder of the patients, who never smoked, was assigned to the never-smoker periodontitis group (*n* = 30). Additionally, 30 individuals who had clinically healthy gingiva and no clinical attachment loss (≤3 mm periodontal probing depth) were included as the healthy group.

Informed consent was obtained from all participants enrolled in the study. Then, the participants were screened clinically, biochemically, and biophysically to exclude individuals with any systemic illnesses. The following exclusion criteria were also used: (1) age younger than 25 or older than 55 years old; (2) fewer than twenty-two permanent teeth; (3) the chronic use or use in the last two weeks of any type of medication; (4) the presence of any chronic medical condition, including diabetes or viral, fungal or bacterial infections; (5) the presence of any medical condition within the previous two weeks, including flu, upper respiratory infections, allergies, skin disorders, or sinus problems; (6) any form of physical trauma experienced within the previous two weeks; (7) the presence of aggressive periodontitis, periodontal abscess, necrotizing ulcerative gingivitis or periodontitis; (8) periodontal treatment and/or antibiotic therapy received within the preceding three months; (9) any type of dental work or tooth extraction(s) performed in the last two weeks; (10) active carious lesions; (11) former smokers, who had quit smoking; and (12) refusal to sign the consent form.

### 2.2. Clinical Periodontal Examination

The patient's medical history was recorded, based on a written questionnaire and 20 to 30 minutes of interviews. For each patient, a set of complete examinations of intraoral full-mouth clinical parameters and the individual number of teeth present (excluding the third molars) was documented. One clinical examiner (SA) performed all of the clinical measurements. Calibration exercises for probing measurements were performed in five patients before the actual study. The intraexaminer agreement was good, with a *k* value of 0.82. The periodontal probing depth (PPD) and clinical attachment level (CAL) were measured at the mesial, distal, buccal, and lingual aspects of each tooth. Smoking history was assessed according to a standardized interview and a self-reported questionnaire. Smoking exposure was expressed in terms of consumption (number of cigarettes per day) and duration (in years).

### 2.3. Collection of GCF Samples

For GCF sampling, teeth numbers 3, 9, 19, and 25 were chosen for both the healthy and periodontitis groups. If one of the participants was missing one of these teeth, then the nearest tooth was used for sampling. Prior to GCF sampling, supragingival plaque was removed from the interproximal surfaces using a sterile curette, and the tooth was gently dried using an air syringe. The area was carefully isolated to prevent samples from being contaminated by saliva. Care was taken to avoid mechanical injury of the gingival tissues. The GCF samples were collected by placing a microcapillary pipette at the entrance of the gingival sulcus, gently touching the gingival margin [23]. The collected GCF samples were immediately transferred to airtight plastic vials and were stored at −70°C until analysis. From each group, a standardized volume of 1 *μ*L was collected, using the calibration on the white, color-coded, 1–5 *μ*L, volumetric microcapillary pipettes (Sigma-Aldrich, St. Louis, MO, USA).

### 2.4. Collection of Saliva Samples

Approximately 2 mL of unstimulated whole saliva was collected in a sterile container using the spit-out method. The saliva was collected 5 minutes after rinsing the mouth thoroughly with distilled water. The saliva samples were kept on ice for an hour. The supernatant (middle 1/3) was collected by centrifugation at 3800 rpm for 10 min and stored at −70°C until analysis.

### 2.5. Estimation of HGF Levels

The concentration of HGF was determined using an ELISA kit (human HGF immunoassay, Quantikine; R and D Systems Inc., Minneapolis, MN). The microplate was precoated with a murine monoclonal antibody specific for HGF. After dilution of the GCF and saliva samples, a 150 *μ*L assay diluent was added to each well with 50 *μ*L of the samples and incubated for 2 hours at room temperature. After washing away any unbound substances, a 200 *μ*L HGF conjugate, an enzyme-linked polyclonal antibody specific for HGF conjugated to horseradish peroxidase with preservative, was added to the wells. Following four washes to remove any unbound antibody-enzyme reagent, a substrate solution was added to the wells. After incubation for 30 minutes, color developed in proportion to the amount of HGF bound during the initial step. The intensity of the color was measured using a microplate reader set to 450 nm. The concentration of HGF in the tested samples was estimated using the reference calibrated standard curve, obtained by plotting the optical density values of the standards against the concentrations [16].

### 2.6. Statistical Analysis

Statistical analysis of the data was performed using the GraphPad InStat software (InStat, GraphPad InStat, Inc., San Diego, CA). Means and standard deviations for age, number of teeth, plaque index, bleeding on probing, periodontal probing depths, clinical attachment levels, and the salivary and GCF HGF levels of the participants (healthy, never-smoker periodontitis, and smoker periodontitis) were analyzed. Differences between the three study groups for all variables were determined with one-way analysis of variance (ANOVA). When an overall ANOVA showed statistical significance, post hoc testing (Tukey-Kramer multiple comparisons test) was performed to explore the differences between any two groups. *P*-values <0.05 were considered significant. Student's *t-*test was used to analyze the mean differences in periodontal probing depth and clinical attachment level between the two periodontitis groups.

## 3. Results

The distribution of subjects in the three groups is presented in Table 1. The periodontal probing depth and clinical attachment levels were measured at six sites, and the mean values were calculated for each individual in the healthy, smoker and never-smoker groups with periodontitis (Table 1). The smoker group had a slightly higher PPD and CAL than the never-smoker periodontitis group, but this difference was not statistically significant.

**Figure 1:**
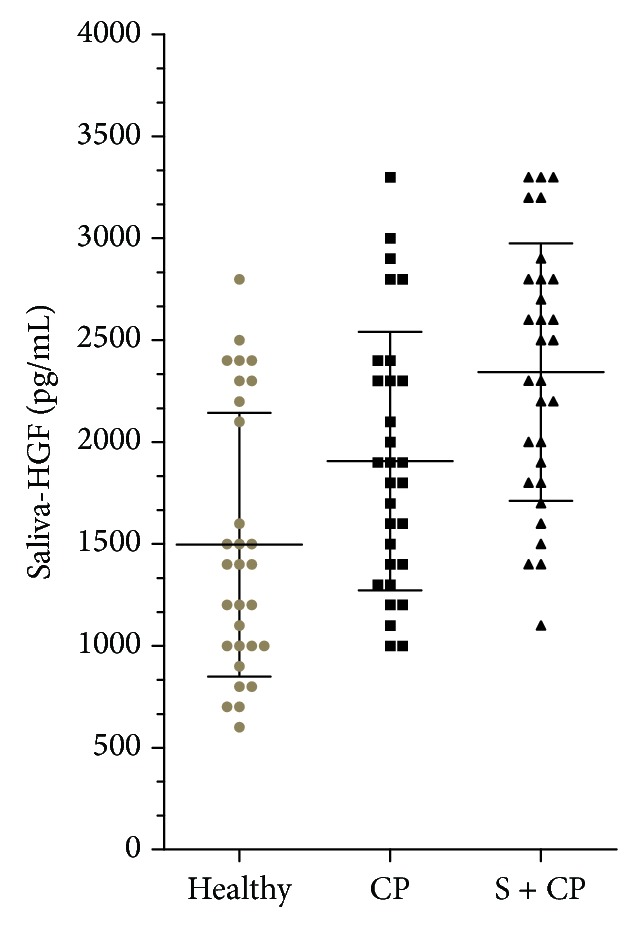
Salivary hepatocyte growth factor levels in periodontally healthy subjects (Healthy), never-smokers with chronic periodontitis (CP) and smokers with periodontitis (S + CP).

**Figure 2:**
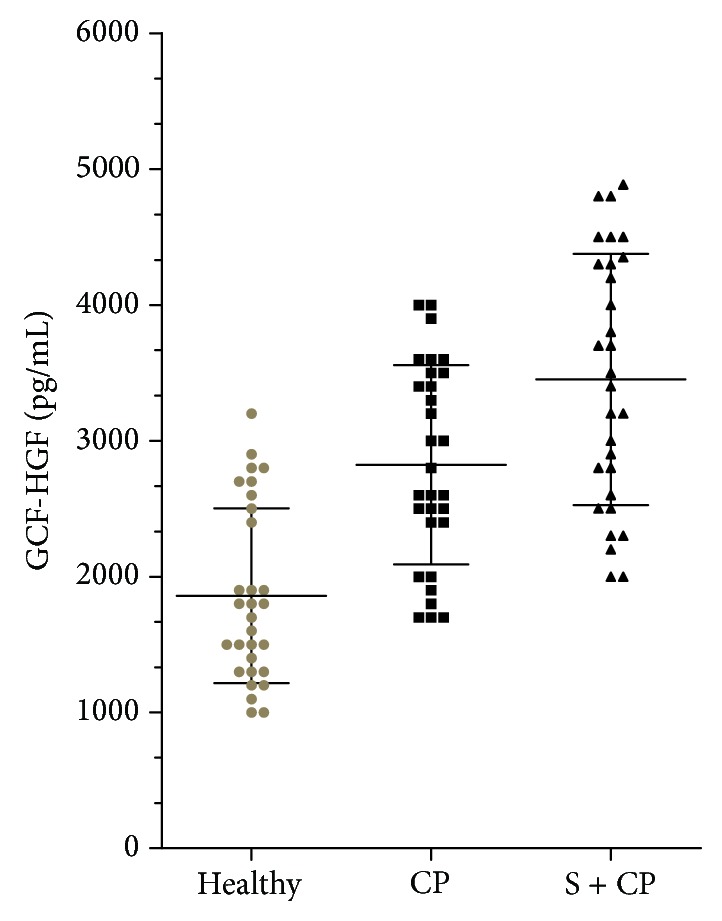
GCF hepatocyte growth factor levels in periodontally healthy subjects (Healthy), never-smokers with chronic periodontitis (CP) and smokers with periodontitis (S + CP).

The salivary HGF was compared among the healthy, never-smoker with periodontitis, and smoker with periodontitis groups. The smoker with periodontitis group had significantly higher levels (*P* < 0.01) of salivary hepatocyte growth factor than the healthy and never-smoker with periodontitis groups (Table 1 and Figure 1). The HGF levels in the GCF also showed a similar pattern (Table 1 and Figure 2), with the highest levels in smokers (3367.86 ± 897.34), followed by nonsmoking subjects with periodontitis (2823.33 ± 733.76) and healthy subjects (1860.00 ± 643.59).

## 4. Discussion

The development of innovative diagnostic tests to detect active phases of periodontal disease and to identify individuals at high risk for future disease occurrence is the focus of numerous clinical investigations. With the advent of highly sensitive techniques, traces of markers can be accurately established in body fluids, such as saliva and GCF [24, 25]. Oral fluids contain locally and systemically derived mediators of periodontal disease, including pathogens, host-response, and bone-specific markers. Most biomarkers in GCF and saliva are indicators of inflammatory events that precede the destruction of the alveolar bone [26].

Hepatocyte growth factor (HGF), a pluripotent, ubiquitous, and mostly regenerative cytokine, is strongly involved in the pathogenesis and progression of periodontitis. HGF stimulates the growth of gingival epithelial cells into the periodontal pockets, thereby impairing the regeneration of the collagenous structures of the periodontium [27]. HGF production is induced by various factors derived from the host, such as inflammatory cytokines, and by the bacterial components. The pathogenic microorganism* Porphyromonas gingivalis* is associated with periodontitis and stimulates the production of HGF by gingival fibroblasts [28]. HGF is produced in the gingiva, and its level in GCF is almost 10-fold higher than that in the serum [17].

Studies have demonstrated elevated HGF levels in patients with various chronic diseases [29, 30]. A high level of HGF in the GCF was reported in patients with periodontitis [16–18]. In periodontitis, the production of HGF is induced by various factors derived from the host, such as inflammatory cytokines and bacterial components. Studies have shown a correlation between the number of deep periodontal pockets and the salivary levels of HGF [31, 32]. In the present study, a significantly higher level of salivary HGF was found in smokers with periodontitis and nonsmokers with periodontitis compared to the control group. These observations corroborate with previous* in vitro* and* in vivo* investigations, indicating the link between HGF and periodontal disease [15, 16, 33].

HGF plays an important role in mesenchymal epithelial interactions, which contributes to wound healing. HGF expression by oral mucosa fibroblasts is elevated compared with the expression by dermal fibroblasts [34]. During inflammatory conditions, such as periodontitis, HGF production by oral fibroblasts is enhanced by various molecules that are induced during inflammation [35]. HGF also enhances the production of matrix metalloproteinase by keratinocytes derived from the oral mucosa [36]. Because c-Met is expressed in many epithelial cells, the mitogenic activity of HGF on epithelial cells, including gingival keratinocytes, is known to play a role in wound healing. Hence, the HGF levels in the GCF and saliva increase substantially due to the overall burden from smoking and bacterial load. The observations from the current study reveal that the estimation of HGF in the crevicular fluid and saliva could serve as a noninvasive indicator of periodontitis. Further studies may help establish the association between the severity of periodontitis and the HGF levels in saliva and GCF.

## 5. Conclusion

Within the limitations of the study, the estimation of HGF in saliva and GCF can be used as a marker of active periodontitis. The significantly higher levels of HGF in smokers with periodontitis might be due to the adverse effects of smoking on the periodontium.

## Figures and Tables

**Table 1:** Study population showing age, clinical attachment level (CAL), pocket depth (PPD), and salivary and GCF concentrations of HGF (Mean ± SD).

Groups (*n* = 30)	Age (years)	PPD (mm)	CAL (mm)	Salivary HGF (pg/mL)	GCF-HGF (pg/mL)
	Mean ± SD	Mean ± SD	Mean ± SD	Mean ± SD	Mean ± SD
Healthy	34.73 ± 6.37	0	0	1496.67 ± 647.27	1860.00 ± 643.59
Never-Smokers with periodontitis	34.67 ± 7.62	5.54 ± 0.34	3.86 ± 0.66	1906.67 ± 634.60	2823.33 ± 733.76
Smokers with periodontitis	34.93 ± 7.97	5.56 ± 0.44	3.90 ± 0.73	2343.33 ± 632.28	3367.86 ± 897.34
